# Methylation Analysis in Monozygotic Twins With Treatment-Resistant Schizophrenia and Discordant Responses to Clozapine

**DOI:** 10.3389/fpsyt.2021.734606

**Published:** 2021-09-20

**Authors:** Masataka Kikuchi, Takanobu Nakazawa, Makoto Kinoshita, Hidenaga Yamamori, Yuka Yasuda, Michiko Fujimoto, Ryota Hashimoto, Shusuke Numata

**Affiliations:** ^1^Department of Genome Informatics, Graduate School of Medicine, Osaka University, Osaka, Japan; ^2^Department of Bioscience, Tokyo University of Agriculture, Tokyo, Japan; ^3^Department of Psychiatry, Institute of Biomedical Sciences, Tokushima University Graduate School, Tokushima, Japan; ^4^Department of Pathology of Mental Diseases, National Center of Neurology and Psychiatry, National Institute of Mental Health, Tokyo, Japan; ^5^Department of Psychiatry, Graduate School of Medicine, Osaka University, Osaka, Japan; ^6^Japan Community Health Care Organization Osaka Hospital, Osaka, Japan; ^7^Medical Corporation Foster, Osaka, Japan

**Keywords:** schizophrenia, clozapine, DNA methylation, monozygotic twins, iPS cells

## Abstract

Schizophrenia is a mental illness that involves both genetic and environmental factors. Clozapine, an atypical antipsychotic, is a well-established therapy for treatment-resistant schizophrenia. In this study, we focused on a set of monozygotic twins with treatment-resistant schizophrenia in which one twin effectively responded to clozapine treatment and the other did not. Our previous study generated neurons from induced pluripotent stem (iPS) cells derived from these patients and compared the transcriptome profiles between mock- and clozapine-treated neurons. In this study, we performed genome-wide DNA methylation profiling to investigate the mechanisms underlying gene expression changes. First, we extracted the differentially methylated sites from each twin based on statistical analysis. Then, we combined the DNA methylation profiling with transcriptome profiling from our previous RNA-seq data. Among the genes with altered methylation and expression, we found the different proportions of the genes related to neuronal and synaptic functions between the clozapine responder and non-responder (35.7 and 6.7%, respectively). This trend was observed even when the basal differences between the responder and non-responder was excluded. These results suggest that effective clozapine action may correct the abnormalities of neuronal and synapse functions in schizophrenia via changes in methylation.

## Introduction

Schizophrenia is characterized by positive symptoms, negative symptoms, and disturbances in basic cognitive functions. Its lifetime prevalence is 0.30–0.66% (1, 2). Approximately 10–30% of patients with schizophrenia show little or no improvement in symptoms after multiple trials of monotherapy (3). Clozapine, which is an atypical antipsychotic drug, is considered for patients with such treatment-resistant schizophrenia. However, the molecular mechanism of action of clozapine is still not fully understood. Several issues should be addressed to uncover its mechanisms. One is the difficulty of obtaining brain tissues from living patients receiving clozapine. Another is the differences in the genetic backgrounds of patients. To overcome these challenges, we focused on a case of monozygotic twins. In this case, both of the identical twins exhibited treatment-resistant schizophrenia, but one twin responded well to clozapine treatment whereas the other twin did not. We had previously established induced pluripotent stem (iPS) cell lines from immortalized B cells from each patient and differentiated neurons from these iPS cells (4). We treated the iPS neurons from each twin with a mock treatment or clozapine, examined the genes expressed in response to clozapine by RNA-seq analysis, and identified differentially expressed genes (DEGs) specific to the clozapine responding twin. However, it remained unclear whether clozapine directly affects gene expression because changes in gene expression are closely related to epigenetic changes such as DNA methylation.

DNA methylation is an epigenetic change, that is, an acquired change in gene regulation that does not involve a change in DNA sequence. DNA methylation is more likely to occur at CpG sites, that is, 5′-CG-3′ dinucleotide sequences, in the genome. DNA methylation is associated with transcriptional regulation, but the relationship is not necessarily the same for gene promoters and gene bodies. The hypermethylation of CpG sites in a promoter is negatively correlated with transcription (5). In contrast, DNA methylation is abundant in the bodies of actively transcribed genes (6–8). In other words, DNA methylation in gene bodies is positively correlated with transcription; therefore, its role is not related to gene silencing (6, 9, 10). Aberrant DNA methylation has been reported in psychiatric disorders, including schizophrenia (11–16). Interestingly, a recent study found significant methylation changes in patients with schizophrenia, especially in treatment-resistant schizophrenia, reflecting the effects of clozapine (17). Additionally, several studies reported that clozapine altered DNA methylation levels in certain genes (18–21). This observation suggests that clozapine may act not only through direct modulation of gene expression but also through epigenetic changes.

In this study, we attempted to obtain a comprehensive DNA methylation profile of iPS neurons obtained from a pair of monozygotic twins with treatment-resistant schizophrenia and discordant responses to clozapine (one a responder and the other a non-responder), and we examined the changes in DNA methylation following clozapine treatment in each patient. Then, we investigated whether DNA methylation was involved in the differential expression of the genes identified in our previous RNA-seq data as exhibiting distinct responses to clozapine treatment.

## Methods

### Subjects

Monozygotic twin patients with treatment-resistant schizophrenia were recruited at Osaka University Hospital. The details of the patients were previously described (4). The twins were 59-year-old Japanese females, both diagnosed with treatment-resistant schizophrenia. Each subject was diagnosed and assessed by at least two trained psychiatrists according to the Diagnostic and Statistical Manual of Mental Disorders, fourth edition (DSM-IV) criteria based on a structured clinical interview. Written informed consent was obtained from subjects after the procedures were fully explained.

### Generation of iPS Cells and *in vitro* Differentiation of iPS Cells Into Excitatory Neurons

The generation of iPS cells from lymphoblastoid B-cell lines and *in vitro* differentiation of iPS cells into excitatory neurons were performed as previously described (4). In brief, immortalized lymphoblastoid B cells were electroporated with plasmids encoding hOCT3/4, hSOX2, hKLF4, hL-MYC, hLIN28 and dominant negative mTP53. After electroporation, cells were seeded onto SNL feeder cells and grown to form colonies. Subsequently, colonies similar to human ES cells were clonally isolated, morphologically selected and evaluated for the expression of pluripotency markers. For *in vitro* differentiation, iPS cells were transferred under feeder-free conditions onto Matrigel (Corning)-coated dishes and cultured in the chemically defined medium Essential 8 (Thermo Fisher Scientific) according to the manufacturer's instructions. The *in vitro* differentiation of iPS cells into neurons through neurogenin2 (Ngn2) overexpression was performed essentially according to Zhang et al. (22). On day 8, 1 μM clozapine (Sigma-Aldrich) or mock treatment was added to the culture medium. After 7 days, Ngn2-induced neurons were harvested for DNA isolation. The gene expression levels in iPS neurons measured by RNA-seq and related statistics were obtained from our previous study (4). In our previous study, total RNA was isolated from three clones obtained from each patient and equal amounts of total RNAs from each patient were combined and sequenced using the Illumina HiSeq2000 system (BGI, Beijing, China) (4). The reads were aligned to the human reference genome hg19. Identifying of DEGs between two samples was performed based on a Poisson distribution (BGI, Beijing, China). The calculated *p*-values were adjusted using FDR q-value to correct for multiple testing. Genes were identified as differentially expressed between the mock and clozapine treatments or between responder and non-responder at a significance level of FDR q-value < 0.05.

### DNA Methylation Analysis

Genomic DNA was extracted from neuronal nuclei using a Blood & Cell Culture DNA kit (Qiagen). We used the same three clones of each twin as those analyzed by our previous RNA-seq analysis (4) for methylation analysis. Bisulfite conversion of 500 ng of genomic DNA was performed with the EZ DNA methylation kit (Zymo Research). DNA methylation levels were assessed according to the manufacturer's instructions using Infinium® HumanMethylation450 BeadChips (Illumina Inc., San Diego, CA, USA), which enable the examination of DNA methylation status at 485,577 CpG sites, and the resulting data were analyzed using the methylation analysis module within the BeadStudio software (Illumina Inc.). The data were mapped to the hg19 genome. The regions described as TSS200 and TSS1500 in this annotation file were defined as the promoter regions. For methylation analysis, IDAT files were processed using the R software package minfi (23). The raw signal data were normalized by the preprocessQuantile function. The methylation status of each CpG site was represented as a β-value that ranged from 0 (completely unmethylated) to 1 (fully methylated). We analyzed 341,629 sites that satisfied the following criteria: (1) β-values with detection *p*-values < 0.01; (2) CpGs with probes having <3 beads; (3) no probe single nucleotide polymorphisms (SNPs) with minor allele frequencies (MAFs) ≥ 1% in the HapMap-JPT population; (4) no probe cross-reactivity; and (5) no SNPs at CpG sites and single-base extension sites. A list of probes and their corresponding MAF values in the Japanese population was derived from Okamura et al. (24). We excluded sites with SNPs and in cross-reactive regions based on a list derived from Chen et al. (25). We performed dmpFinder function in the minfi package to identify positions that were differentially methylated. Methylation sites were identified as differentially methylated between the mock and clozapine treatments or between responder and non-responder at a significance level of *p*-value < 0.01. The calculated *p*-values were adjusted using FDR q-value to correct for multiple testing, although there were no methylation sites differentially methylated between the mock and clozapine treatments at a significance level of FDR q-value < 0.05.

### The Direction of Change for the mRNA Expression Level and the DNA Methylation Level of the DEGs

The same direction means that both the mRNA expression level and the DNA methylation level are higher or lower in clozapine compared to mock treatment. The opposite direction indicates highly express and lower methylate in clozapine compared to mock treatment, or vice versa.

### Genes Associated With Neuronal and Synaptic Functions

Genes associated with neuronal and synaptic functions were defined as those genes annotated with Gene Ontology terms including the following words: “neuro,” “synapse,” or “synaptic.” We used the information from the Gene Ontology project (http://geneontology.org/) and R software package GO.db. We obtained 2,756 genes after the procedures. Furthermore, we selected 2,399 genes overlapped with 15,466 genes that we treated in the RNA-seq and DNA methylation analyses.

### Gene Functional Enrichment Analysis

Gene functional enrichment analysis was performed using Metascape software (26). Metascape reports a term that an input gene list overrepresented as an enrichment cluster and prevents redundancies in terms across different ontology sources.

### Statistical Analysis

To examine the extent of overlap between the DEGs and the genes related to neuronal and synaptic functions, we calculated the *p*-value by hypergeometric distribution test and the expected numbers as follows:


(1) $$\begin{array}{l} P\left(X=x\right)=\frac{\left(\frac{m}{x}\right)\left(\frac{N-m}{n-x}\right)}{\left(\frac{N}{n}\;\right)}, \end{array}$$

(2) $$\begin{array}{l} Expected=\;\frac{mn}{N}, \end{array}$$

where *x* is the number of genes that overlapped between the DEGs and the genes related to neuronal and synaptic functions, and *m* and *n* are the numbers of the DEGs and the genes related to neuronal and synaptic functions (2,399 genes), respectively. *N* is the total number of genes (15,466 genes that we treated in the RNA-seq and DNA methylation analyses).

## Results

### Changes in DNA Methylation Following Clozapine Treatment in iPS Neurons From Twins With Discordant Responses to Clozapine

**Figure 1:**
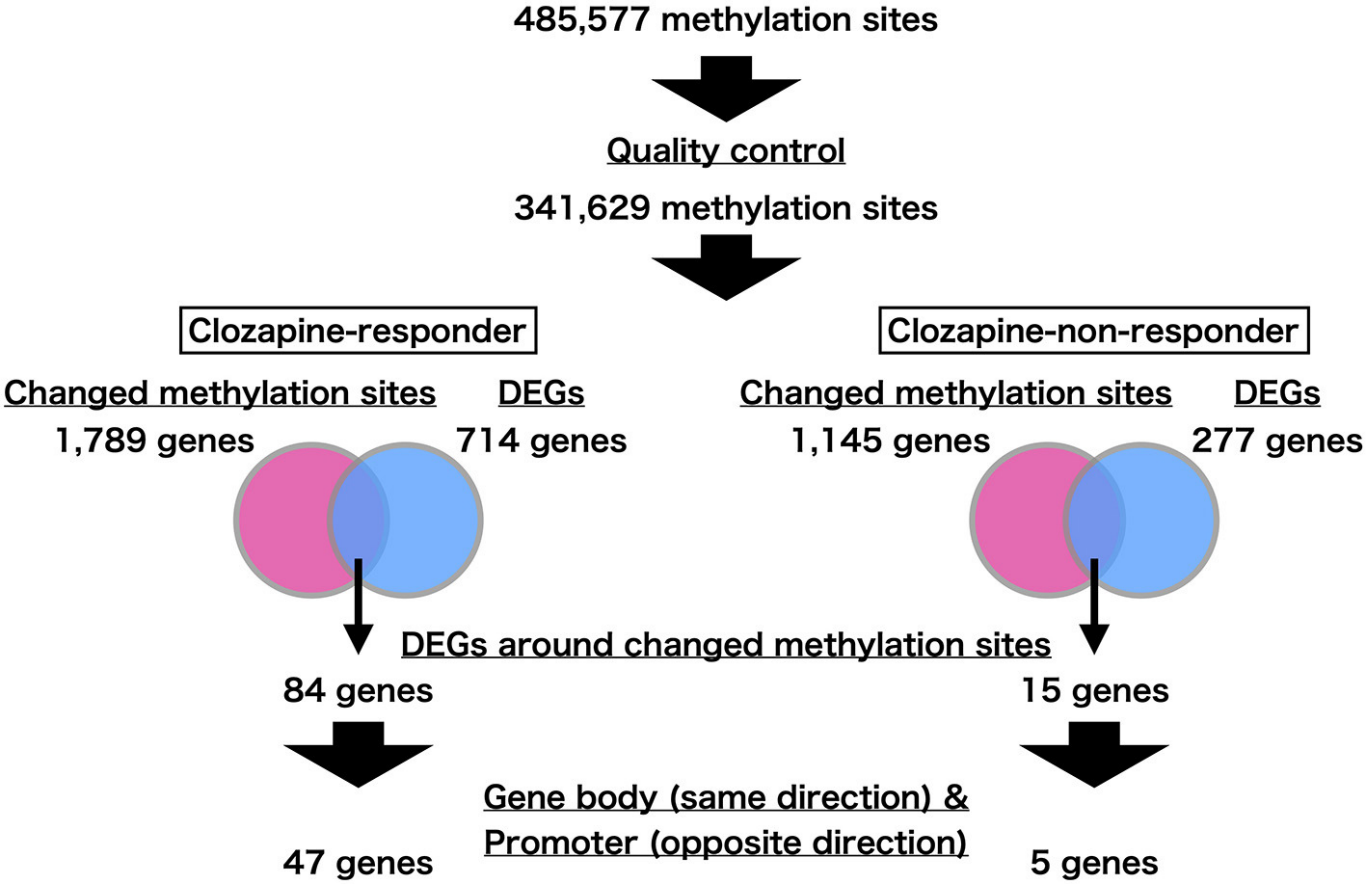
Flowchart of the present study. We first found the genes near the changed methylation sites. We next extracted differentially expressed genes (DEGs) from this set based on transcriptome profiling. Focusing on the methylation sites in gene bodies and promoter regions, we identified genes whose expression changes were consistent with the methylation changes.

We show an overview of the present study in Figure 1. We performed genome-wide DNA methylation profiling using differentiated neurons from iPS cell clones obtained from clozapine responders and non-responders. We analyzed 341,629 of 485,577 methylation sites based on robustness criteria (see section Methods). To identify positions that were differentially methylated following clozapine treatment, we compared the DNA methylation levels of each methylation site between the mock and clozapine-treated neurons in each twin. We extracted the changed methylation sites from each comparison at a significance level of *p*-value < 0.01. As a result, we identified 2,229 and 1,384 methylation sites in the clozapine responder and non-responder, respectively (Supplementary Tables 1, 2). Among these changed methylation sites, only 21 sites were overlapped between the twins. After we excluded the 21 overlapped sites from the changed methylation sites in each twin, these patient-specific methylation sites were involved 1,789 and 1,145 genes in the clozapine responder and non-responder, respectively. We used these methylation data in the subsequent analyses.

### Combined Transcriptome Profiling With DNA Methylation Profiling

We recently identified DEGs following clozapine treatment in iPS neurons from the same twins by performing RNA-seq analysis (4). We found 714 DEGs specific to the clozapine responder and 277 DEGs specific to the clozapine non-responder (FDR q-value < 0.05). Among 714 DEGs identified in the clozapine responder, 84 genes were located near the responder-specific methylation sites (Supplementary Table 3). Of the 84 DEGs, 17 DEGs, including *MECP2* (methyl-CpG-binding protein 2), which has been implicated in schizophrenia and autism as well as Rett syndrome (27, 28), differed in the opposite direction of the change in the methylation sites in the promoter regions (Table 1; Supplementary Figure 1), and 32 DEGs showed the same direction of change for the mRNA expression level and the DNA methylation level in the corresponding gene body (Table 2). Of the DEGs associated with the promoters and gene bodies, 2 genes [*STOX2* (storkhead box 2) and *TBC1D16* (TBC1 domain family member 16)] were common. Among 277 DEGs identified in the clozapine non-responder, 15 genes were located near the non-responder-specific methylation sites (Supplementary Table 4). Of these 15 DEGs, 2 DEGs differed in the opposite direction of the change in the methylation sites in the promoter regions (Table 1), while 3 DEGs showed the same direction of change in the mRNA expression level and the DNA methylation level in the corresponding gene body (Table 2).

**Table 1:** Genes changed in the opposite direction to promoter CpG sites in response to clozapine.

			**Responder**		**Non-responder**	
**CpG probe**	**Gene**	**Description**	**Log2 ratio of methylation**	**Log2 ratio of mRNA**	**Log2 ratio of methylation**	**Log2 ratio of mRNA**
**Responder**						
cg02025573	KIAA0100	KIAA0100	0.157	−0.260	0.023	−0.032
cg06012574	ANKRD11	ankyrin repeat domain 11	0.239	−0.285	−0.009	0.010
cg06502510	ITSN1	intersectin 1	0.276	−0.368	−0.246	0.030
cg08463298	EIF4G3	eukaryotic translation initiation factor 4 gamma 3	0.101	−0.239	−0.082	0.030
cg10232470	TBC1D16	TBC1 domain family member 16	0.389	−0.324	0.001	−0.073
cg12989020	ARID1B	AT-rich interaction domain 1B	0.171	−0.252	0.120	0.016
cg13189687	MECP2	methyl-CpG binding protein 2	0.047	−0.341	−0.001	0.070
cg16010370	TKT	transketolase	−0.303	0.151	0.078	−0.018
cg18165031	ERH	enhancer of rudimentary homolog	−0.456	0.266	−0.195	0.062
cg20016416	SPTBN2	spectrin beta, non-erythrocytic 2	0.007	−0.272	0.010	−0.043
cg20465219	PSMA5	proteasome subunit alpha 5	−0.207	0.312	−0.001	−0.064
cg20715295	PAFAH1B3	platelet activating factor acetylhydrolase 1b catalytic subunit 3	−0.064	0.155	−0.004	0.068
cg24798305	SMC1A	structural maintenance of chromosomes 1A	0.034	−0.244	−0.002	0.089
cg25419628	NHLRC2	NHL repeat containing 2	0.223	−1.077	−0.190	0.350
cg25669309	STOX2	storkhead box 2	0.734	−0.844	−0.316	−0.016
cg27489994	TPT1	tumor protein, translationally-controlled 1	−0.238	0.101	−0.091	0.062
cg27663249	SRRM2	serine/arginine repetitive matrix 2	0.020	−0.221	0.022	−0.072
**Non-responder**						
cg00828305	HNRNPA3	heterogeneous nuclear ribonucleoprotein A3	0.063	0.026	−0.222	0.167
cg19561607	BCAT1	branched chain amino acid transaminase 1	−0.029	−0.030	−0.020	0.202

*Log2 ratio was calculated as the mean value in clozapine treatment divided by the mean value in mock treatment*.

**Table 2:** Genes changed in the same direction as the gene body CpG sites in response to clozapine.

			**Responder**		**Non-responder**	
**CpG probe**	**Gene**	**Description**	**Log2 ratio of methylation**	**Log2 ratio of mRNA**	**Log2 ratio of methylation**	**Log2 ratio of mRNA**
**Responder**						
cg00831127	EPHB2	EPH receptor B2	−0.040	−0.347	−0.005	0.087
cg01210622	SMPD3	sphingomyelin phosphodiesterase 3	−0.009	−0.165	−0.002	0.038
cg02078626	PTPRF	protein tyrosine phosphatase, receptor type F	−0.021	−0.176	−0.012	−0.136
cg04919489	ARHGEF12	Rho guanine nucleotide exchange factor 12	−0.018	−0.262	0.008	0.073
cg04983933	CACNA1H	calcium voltage-gated channel subunit alpha1 H	−0.007	−0.245	−0.005	−0.084
cg05966923	CACNA1H	calcium voltage-gated channel subunit alpha1 H	−0.017	−0.245	0.006	−0.084
cg06608374	KIAA1244	KIAA1244	−0.013	−0.366	0.001	0.082
cg08230332	BSN	bassoon presynaptic cytomatrix protein	−0.428	−0.309	0.244	−0.039
cg08264906	EPHB2	EPH receptor B2	−0.045	−0.347	0.006	0.087
cg08583240	MAP4K4	mitogen-activated protein kinase kinase kinase kinase 4	−0.022	−0.124	−7.67.E-05	0.050
cg08959305	TMEM97	transmembrane protein 97	0.278	0.282	0.133	0.035
cg10399269	CACNA1H	calcium voltage-gated channel subunit alpha1 H	−0.015	−0.245	0.018	−0.084
cg10688790	CNTNAP2	contactin associated protein-like 2	−0.033	−0.188	0.018	−0.092
cg13023584	PCDHGA4	protocadherin gamma subfamily A, 4	−0.014	−2.459	0.003	0.322
cg13023584	PCDHGB4	protocadherin gamma subfamily B, 4	−0.014	−0.254	0.003	−0.060
cg13226797	GAB2	GRB2 associated binding protein 2	−0.012	−0.196	0.006	0.005
cg13406243	CACNA1B	calcium voltage-gated channel subunit alpha1 B	−0.050	−0.175	0.018	0.030
cg13702357	HIP1	huntingtin interacting protein 1	−0.204	−0.246	0.155	0.082
cg14634760	BAI1	Brain-specific angiogenesis inhibitor 1	−0.033	−0.219	0.012	−0.124
cg14723566	ARNT2	aryl hydrocarbon receptor nuclear translocator 2	−0.013	−0.190	0.007	−0.033
cg15127832	STOX2	storkhead box 2	−0.015	−0.844	−0.002	−0.016
cg15379837	TNRC18	trinucleotide repeat containing 18	−0.012	−0.367	0.002	−0.172
cg17879478	STK35	serine/threonine kinase 35	−0.014	−0.328	0.010	−0.050
cg18380070	AGRN	agrin	−0.019	−0.122	0.011	−0.023
cg21459532	RNF152	ring finger protein 152	−0.014	−0.693	0.008	−0.004
cg21532659	NAV2	neuron navigator 2	−0.379	−0.370	−0.088	0.001
cg21623179	TBC1D16	TBC1 domain family member 16	−0.012	−0.324	−0.004	−0.073
cg21627409	PCDHGA4	protocadherin gamma subfamily A, 4	−0.016	−2.459	0.012	0.322
cg21627409	PCDHGB4	protocadherin gamma subfamily B, 4	−0.016	−0.254	0.012	−0.060
cg23323297	SHANK1	SH3 and multiple ankyrin repeat domains 1	−0.041	−0.494	0.036	−0.034
cg23912522	MEG3	maternally expressed 3	0.014	0.376	−0.010	0.171
cg24455683	IQCE	IQ motif containing E	−0.019	−0.364	−0.003	−0.136
cg24471980	RERE	arginine-glutamic acid dipeptide repeats	−0.015	−0.236	−0.009	−0.124
cg24686236	TRIO	trio Rho guanine nucleotide exchange factor	−0.012	−0.306	0.001	0.079
cg24902478	NAV3	neuron navigator 3	−0.007	−0.445	−0.003	0.124
cg26979107	MAPT	microtubule associated protein tau	−0.025	−0.115	−0.009	0.019
cg27028800	CDC42BPB	CDC42 binding protein kinase beta	−0.020	−0.219	0.003	−0.103
**Non-responder**						
cg00029640	USF2	upstream transcription factor 2, c-fos interacting	−0.001	0.027	−0.017	−0.199
cg04685253	PRR12	proline rich 12	−0.012	−0.146	−0.036	−0.280
cg22518079	SETBP1	SET binding protein 1	−0.001	−0.231	0.031	0.399

*Log2 ratio was calculated as the mean value in clozapine treatment divided by the mean value in mock treatment*.

Then, we examined the proportion of genes associated with neuronal and synaptic functions (Table 3). Among the genes near the changed methylation sites, 300 of the 1,789 genes (16.8%) in the clozapine responder and 190 of the 1,145 genes (16.6%) in the clozapine non-responder were associated with neuronal and synaptic functions. Among the genes with altered methylation and expression, 30 of the 84 genes (35.7%) in the clozapine responder and 1 of the 15 genes (6.7%) in the clozapine non-responder were associated with neuronal and synaptic functions. The number of the genes with neuronal and synaptic functions in the clozapine responder was significantly 2.3 times higher than the expected value (*p*-value = 2.96E-06; #observed genes / #expected genes = 2.30), whereas the number of the genes in non-responder was lower than the expected value (*p*-value = 0.220; #observed genes / #expected genes = 0.430). When we performed gene functional enrichment analysis to investigate the functions of these 84 DEGs, we found axon guidance (FDR q-value = 1.59E-07) and neuron projection morphogenesis (FDR q-value = 1.21E-05) in the gene functional enrichment clusters (Supplementary Figure 2). In contrast, we did not find any statistically significantly enriched function in the clozapine non-responder. Among the genes whose expression changes were consistent with the methylation changes in the gene body or promoter regions, 19 of the 47 genes (40.4%) in the clozapine responder were significantly associated with neuronal and synaptic functions (*p*-value = 2.53E-05; #observed genes / #expected genes = 2.61), while there were no associations in the clozapine non-responder (*p*-value = 0.430; #observed genes / #expected genes = 0.00).

**Table 3:** The proportion of genes associated with neuronal and synaptic functions.

	**Neuronal genes (observed)**	**Corresponding genes**	**Expected**	**Observed/expected**	***P*** **-value**
**Responder**					
Changed methylation sites	300 (16.8%)	1,789	277.5	1.081	0.008
DEGs around changed methylation sites	30 (35.7%)	84	13	2.302	2.96.E-06
Gene body (same direction) and Promoter (opposite direction)	19 (40.4%)	47	7.3	2.606	2.53.E-05
**Non-responder**					
Changed methylation sites	190 (16.6%)	1,145	177.6	1.07	0.019
DEGs around changed methylation sites	1 (6.7%)	15	2.3	0.43	0.22
Gene body (same direction) and Promoter (opposite direction)	0 (0.0%)	5	0.8	0	0.43

*P-values were calculated by hypergeometric distribution test*.

### The Differences of the Basal Methylation States of the Responder and Non-responder

We finally examined the differences of the basal methylation states of the responder and non-responder. We identified 3,065 and 2,980 methylation sites that changed between the twins in the mock and clozapine treatments at a significance level of *p*-value < 0.01, respectively. Among these changed methylation sites, 308 sites were overlapped between the two treatments. Each changed methylation sites were involved 2,305 and 2,241 genes in the mock and clozapine treatments, respectively. The RNA-seq analysis found 1,705 DEGs in the mock treatment and 806 DEGs in the clozapine treatment (FDR q-value < 0.05). Among 1,705 DEGs identified in mock treatment, 133 genes were located near the 3,065 changed methylation sites. Among 806 DEGs identified in the clozapine treatment, 88 genes were located near the 2,980 changed methylation sites. The 133 DEGs in mock treatment were overlapped with only 5 genes among the 84 DEGs response to clozapine in the clozapine responder. Additionally, there were no genes overlapped between 133 DEGs in mock treatment and 15 DEGs in the clozapine non-responder.

## Discussion

We examined the DNA methylation changes following clozapine treatment in iPS neurons from a pair of twins with discordant responses to clozapine. To date, there have been several studies of schizophrenia using human iPS cells (29). Applying iPS cell-based technology may provide new insight into the therapeutic mechanism of clozapine. We demonstrated that there were only a small number of CpG sites that were common between the clozapine responder and non-responder among the changed methylation sites. Then, we combined our previous transcriptome profiling data with DNA methylation profiling to identify genes whose expression changes and methylation changes were consistent. When we examined the correlations between the mRNA expression and DNA methylation levels, we found cases with changes in not only the opposite direction of the promoter but also the same direction in the corresponding gene body. These findings were in accord with recent reports that the bodies of actively transcribed genes are enriched in DNA methylation (6–8). There are two hypotheses about the function of DNA methylation in gene bodies (30). One is that it facilitates transcription elongation and/or co-transcriptional splicing. The other is that it represses intragenic cryptic promoters. Importantly, we demonstrated that genes related to neuronal and synaptic functions were observed at a higher frequency among the genes with altered DNA methylation and expression in the clozapine responder than in the clozapine non-responder. These results suggest that effective clozapine action may normalize the abnormalities of neuronal and synaptic functions via methylation changes.

We revealed increased DNA methylation in the promoter region and decreased expression in the *MECP2* gene following clozapine treatment in the clozapine responder (Supplementary Figure 1). Basic research has shown that MeCP2 plays an important role in mediating synaptic transmission in the CNS (31, 32), and mutations in MECP2 are well-known to be implicated in neurodevelopmental disorders, including schizophrenia (28, 33). MECP2 binds to the promoter regions of methylated genes and suppresses their expression (34, 35). It was reported that MECP2 regulates thousands of genes in the hypothalamus and that 85% of these target genes were transcriptionally activated by MECP2 using comprehensive mRNA expression analysis of MECP2 knockout and MECP2 overexpression mice (36). Our results suggest that clozapine may affect the mRNA expression level of the *MECP2* gene via DNA methylation as well as the transcriptomes of multiple downstream genes.

Among the methylation sites that specifically responded to clozapine in the responder, cg03942932 showed significant methylation changes in a previous meta-analysis study of DNA methylation changes in schizophrenia and controls using blood samples (17). In addition, the responder-specific cg19939130 was also significantly altered between schizophrenia and controls in a study using African American blood samples (14). Interestingly, DOT1L (DOT1 like histone lysine methyltransferase), which was a gene with methylation sites that varied specifically in responders, was reported as the gene with the most variable methylation sites in blood between treatment-resistant and non-treatment-resistant schizophrenic patients (17).

In our previous study, we performed whole exome sequencing analysis of the twins and did not observe any genomic discordance on exons between the twins (4). However, there may be differences in mutations in non-coding regulatory regions such as promoters between the twins, and such mutations may affect methylation. Detailed observations by targeted sequencing will be necessary. If there is no genomic discordance between the twins, the reason for their discordance in clozapine response may be somatic changes during development. We also cannot exclude the possibilities that the differences in clozapine responsiveness observed between the twins' iPS cell-derived neurons are affected by the reprogramming process that can largely reset DNA methylation patterns in somatic cells and/or by the NGN2-memdiated neuronal differentiation. Further studies with neurons directly differentiated from the twins' somatic cells or iPS cell-derived neurons differentiated without viral overexpression of NGN2 would be necessary to validate our findings.

There are several limitations in the present study. As this is a case report with a pair of monozygotic twins, the findings need to be validated in studies with larger cohort including healthy control individuals to demonstrate the disease relevancy. In addition, further *in vivo* experiments and experiments with animal model will be required to reveal how the DNA methylation levels of the CpG sites and mRNA expression levels of the corresponding genes are altered by clozapine in the responder-derived neurons and what determines the responsiveness to clozapine.

## Data Availability Statement

The original contributions presented in the study are included in the article/Supplementary Material, further inquiries can be directed to the corresponding author/s.

## Ethics Statement

The studies involving human participants were reviewed and approved by The Research Ethical Committees of Osaka University and Tokushima University. The patients/participants provided their written informed consent to participate in this study.

## Author Contributions

MKik and SN contributed to the study design and wrote the manuscript. MKik analyzed the data. TN performed the iPS cell experiments. MKin obtained the DNA methylation data. HY, YY, and MF contributed to the data collection. MKik, TN, MKin, HY, YY, MF, RH, and SN contributed text to the manuscript. All authors read and approved the final manuscript.

## Funding

This work was supported by Grants-in-Aid for Scientific Research [grant numbers 17K15049 (MKik), 20K15778 (MKik), 21H02628 (TN), 21H00213 (TN), 20K07945 (MF), JP20H03611 (RH), and JP18KT0022 (RH)] from the Ministry of Education, Culture, Sports, Science and Technology (MEXT); the Health and Labour Sciences Research Grants for Comprehensive Research on Persons with Disabilities [16dk0307065 h001 (TN, MKik, SN, and RH)], JP21gm1310003 (TN), JP21dk0307103 (RH) from the Japan Agency for Medical Research and Development (AMED); a grant from the Takeda Science Foundation (TN); a grant from the Asahi Glass Foundation (TN); a grant from the Naito Foundation (TN); and an Intramural Research Grant (3-1) for Neurological and Psychiatric Disorders of NCNP (RH). The funders had no role in the study design, data collection and analysis, decision to publish, or preparation of the manuscript.

## Conflict of Interest

YY is employed by Medical Corporation Foster. The remaining authors declare that the research was conducted in the absence of any commercial or financial relationships that could be construed as a potential conflict of interest.

## Publisher's Note

All claims expressed in this article are solely those of the authors and do not necessarily represent those of their affiliated organizations, or those of the publisher, the editors and the reviewers. Any product that may be evaluated in this article, or claim that may be made by its manufacturer, is not guaranteed or endorsed by the publisher.
