# Supplementary material for: Methylation Analysis in Monozygotic Twins With Treatment-Resistant Schizophrenia and Discordant Responses to Clozapine
Source: Front Psychiatry. 2021 Sep 20;12:734606. doi: 10.3389/fpsyt.2021.734606 (PMC8488120; doi:10.3389/fpsyt.2021.734606)
Supplement: Supplementary file 2 [file Data_Sheet_1.PDF]

FigureS1

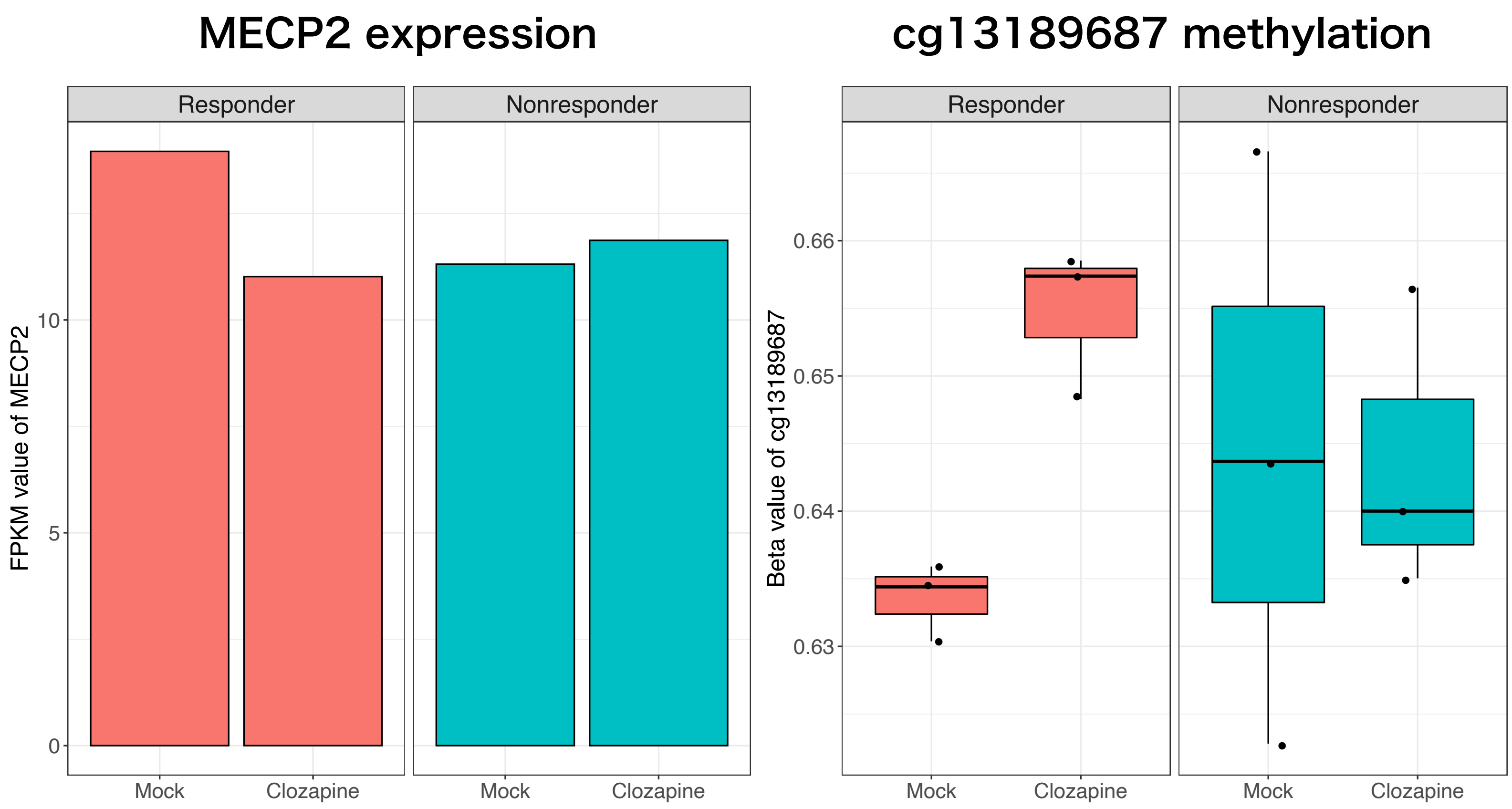

Figure S1. The mRNA expression and methylation levels of MECP2. Left panel shows FPKM value of MECP2 gene by RNA-seq (n=1 in each group). Right panel shows beta value of cg13189687 CpG probe by methylation analysis (n=3 in each group).

FigureS2

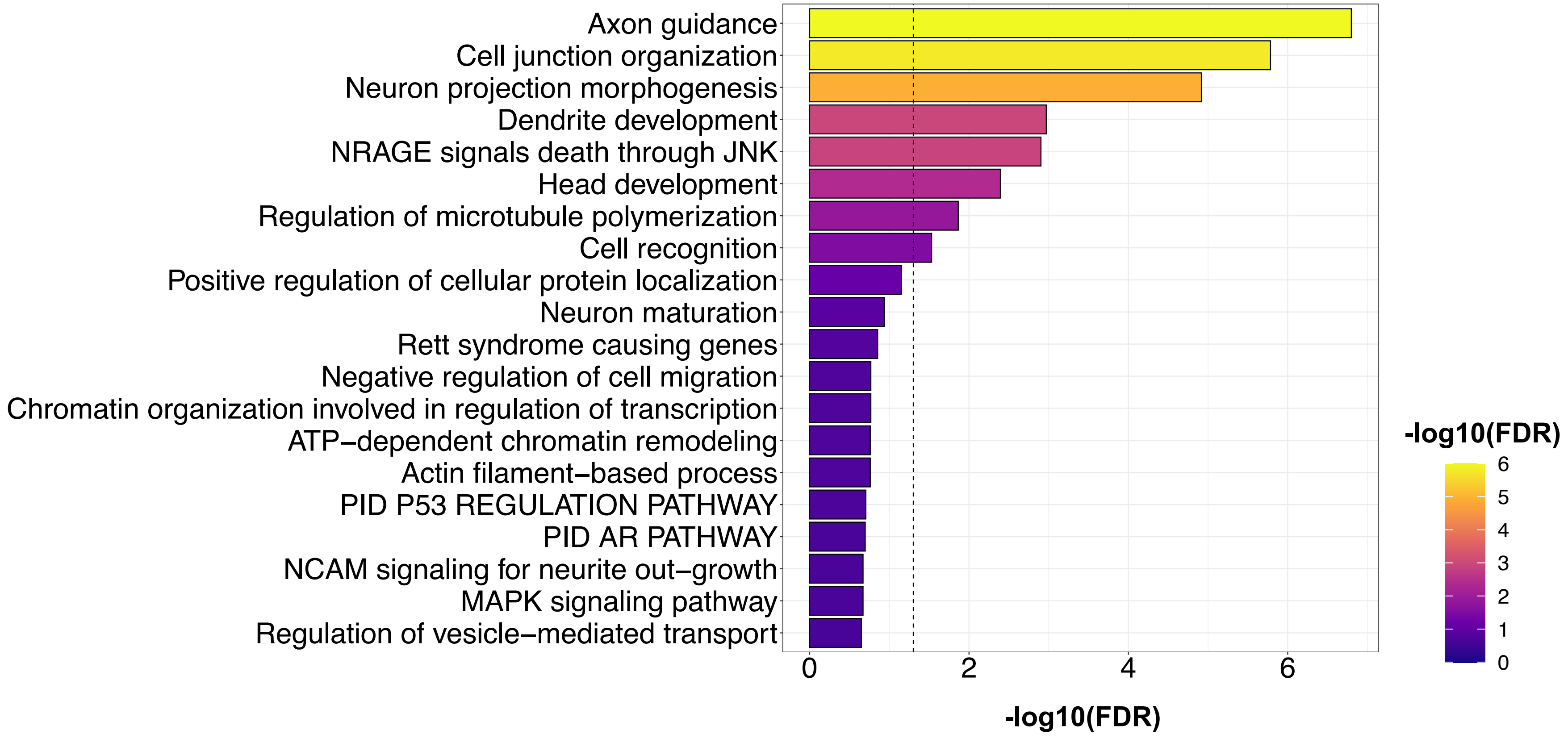

Figure S2. The gene functional enrichment clusters of the clozapine-responder-specific DEGs that were located around the changed methylation sites in the clozapine-responder. Each bar indicates statistical significance calculated by metascape software. Each term shows representative terms in enrichment clusters. A vertical line represents FDR q-value = 0.05.
